# Fluorine-19 Magnetic Resonance Angiography of the Mouse

**DOI:** 10.1371/journal.pone.0042236

**Published:** 2012-07-27

**Authors:** Ruud B. van Heeswijk, Yves Pilloud, Ulrich Flögel, Jürg Schwitter, Matthias Stuber

**Affiliations:** 1 Department of Radiology, Centre Hospitalier Universitaire Vaudois and University of Lausanne, Lausanne, Switzerland; 2 Center for Biomedical Imaging (CIBM), Lausanne, Switzerland; 3 Laboratory for Functional and Metabolic Imaging (LIFMET), Ecole Polytechnique Fédérale de Lausanne (EPFL), Lausanne, Switzerland; 4 Department of Cardiovascular Physiology, Heinrich Heine University, Düsseldorf, Germany; 5 Cardiology Service, Centre Hospitalier Universitaire Vaudois, Lausanne, Switzerland; Cornell University, United States of America

## Abstract

**Purpose:**

To implement and characterize a fluorine-19 (^19^F) magnetic resonance imaging (MRI) technique and to test the hypothesis that the ^19^F MRI signal in steady state after intravenous injection of a perfluoro-15-crown-5 ether (PCE) emulsion may be exploited for angiography in a pre-clinical *in vivo* animal study.

**Materials and Methods:**

*In vitro* at 9.4T, the detection limit of the PCE emulsion at a scan time of 10 min/slice was determined, after which the T_1_ and T_2_ of PCE in venous blood were measured. Permission from the local animal use committee was obtained for all animal experiments. 12 µl/g of PCE emulsion was intravenously injected in 11 mice. Gradient echo ^1^H and ^19^F images were obtained at identical anatomical levels. Signal-to-noise (SNR) and contrast-to-noise (CNR) ratios were determined for 33 vessels in both the ^19^F and ^1^H images, which was followed by vessel tracking to determine the vessel conspicuity for both modalities.

**Results:**

*In vitro*, the detection limit was ∼400 µM, while the ^19^F T_1_ and T_2_ were 1350±40 and 25±2 ms. The ^19^F MR angiograms selectively visualized the vasculature (and the liver parenchyma over time) while precisely coregistering with the ^1^H images. Due to the lower SNR of ^19^F compared to ^1^H (17±8 vs. 83±49, p<0.001), the ^19^F CNR was also lower at 15±8 vs. 52±35 (p<0.001). Vessel tracking demonstrated a significantly higher vessel sharpness in the ^19^F images (66±11 vs. 56±12, p = 0.002).

**Conclusion:**

^19^F magnetic resonance angiography of intravenously administered perfluorocarbon emulsions is feasible for a selective and exclusive visualization of the vasculature *in vivo*.

## Introduction

A considerable research effort is directed at improving the detection of vascular disease with different modalities such as X-ray, intravascular ultrasound (IVUS), computed tomography (CT) and magnetic resonance angiography (MRA) [1]. Because of its non-invasive nature and lack of harmful radiation, MRA is considered the most patient-friendly among these techniques, while soft-tissue characteristics and blood flow can easily be exploited for contrast generation.

The main challenge for MRA is associated with the fact that signal originates from water protons, which are abundant in the entire body. Inevitably, this leads to background signal in the image and the blood-pool cannot be seen exclusively. This means that some strategy is needed to attenuate the signal from static tissue in close proximity to the blood vessels. Contemporary MRA is therefore commonly performed using intravascular [2] or extracellular [3] T_1_-lowering gadolinium (Gd) contrast agents, time-of-flight (TOF) imaging, or phase contrast (PC) imaging.

Instead of lowering the T_1_ relaxation time or using flow characteristics, the frequency of the magnetic resonance imaging (MRI) signal itself can also be shifted and exploited for contrast generation. This should be feasible, since in addition to ^1^H, magnetic resonance imaging can also detect several other nuclei. Fluorine (^19^F) is such a nucleus and when imaged has 85% of the MRI sensitivity of ^1^H, as well as MRI-negligible natural concentrations in the body [4]. When incorporated in perfluorocarbons (PFC, carbohydrates in which all hydrogen has been replaced by fluorine) the results are biochemically inert, non-toxic molecules of which some can be used as blood volume expanders and oxygen carriers, and have been safely and successfully used in several phase 3 trials in human patients [5]. Since PFCs are both hydrophobic and lipophobic, they are usually incorporated in emulsions to allow for a longer circulation time in blood. Such PFC emulsions have already been used for angiography in perfused excised organs such as the heart and kidneys [6], [7], as well as in ‘first-pass’ regional angiography of carotid arteries in rabbits in combination with a gadolinium contrast agent for fast imaging [7].

These non-toxic emulsions can thus be directly injected intravenously and ^19^F MRI of the blood vessels might also be performed once they have spread through the entire vasculature. In a manner similar to off-resonance angiography with iron-oxide nanoparticles [8], the ^19^F signal will only originate from the injected PFC that will remain in the vascular system; no background signal is expected, and direct flow-independent imaging of the blood vessels may be feasible. Furthermore, if no T_1_-lowering gadolinium is added to the injection, the relaxation times T_1_ and T_2_ of the ^19^F agent remain constant, and no adaptations of the pulse sequence parameters as a function of time after injection will be necessary, and anatomy distant from the injection site (such as the heart chambers and great vessels) can also be visualized. Therefore, in this this pre-clinical study in rodents we wanted to develop, characterize and test a novel ^19^F MRI technique *in vitro* to test the hypothesis *in vivo* that the ^19^F MR signal after systemic perfluorocarbon injection may be exploited for angiography with conventional non-contrast ^1^H MRA as a reference.

## Methods

All experiments were performed on a Varian (Palo Alto, CA, USA) 9.4T animal spectrometer. A dedicated custom-built quadrature surface coil with 18-mm diameter loops was used for ^19^F and ^1^H RF transmission and reception. Preparation of a 10% perfluoro-15-crown-5 ether (crown ether or PCE) emulsion was carried out as previously described [9].

To determine its T_1_ and T_2_ in an oxygenated and deoxygenated environment, 0.5 ml of the emulsion was added to 1.4 ml venous blood as well as to 1.4 ml of saline (to simulate oxygenated blood) for a final PCE concentration of 50 mM. An adiabatic inversion-recovery spectroscopy sequence with a repetition time (TR) of 10 s and an inversion time (TI) that increased from 0.001 to 20 s was used for T_1_ determination, while an adiabatic Carr-Purcell-Meiboom-Gill (CPMG) spectroscopy sequence was applied for T_2_ measurements with TR = 10 s and echo times (TE) ranging from 0.004 to 2 s.

Next, a dilution series helped ascertain the detection limit of the MRI pulse sequence within a 10 min measurement time limit. 50 mM PCE in the saline sample described above was diluted to a series with PCE concentrations of 10, 2.0, 0.80 and 0.40 mM. Subsequently, ^19^F gradient echo (GRE) images (30×30 mm^2^ field of view, 64×64 resolution, TR/TE = 5.4/2.5 ms, slice thickness = 3 mm, 1750 averages, 10 min/slice, 2 slices) were acquired and the signal-to-noise ratio (SNR) of the unprocessed images was determined. These *in vitro* images were then postprocessed and thresholded at 90% of the maximum signal intensity to improve detection of weaker signals; the detection limit with the postprocessing was experimentally determined as SNR = 2 in the original images [10].

For the *in vivo* part of these studies, which was approved by the state animal use committee (‘Service de la Consommation et des Affaires Vétérinaires du canton de Vaud’ authorization number 2374), male balb/c mice (11 animals, 28±2 g bodyweight) were anesthetized with isoflurane and a catheter was inserted into their tail vein. ECG leads were attached to their paws and the animal was placed in a dedicated holder on top of the surface coil, centered between the heart and kidneys. Next, 12 µl/g bodyweight of the PCE emulsion was infused through the catheter over ∼3 min.

Non-triggered ^1^H 128×128 TOF-GRE (TR/TE = 6.1/2.8 ms) and 64×64 TOF-GRE (TR/TE = 4.7/2.0 ms) images with a high radiofrequency excititation angle (not quantifiable since it varies with distance from the surface coil) were acquired in the 3 principal directions (field of view 30×30 cm^2^, slice thickness 2 or 3 mm, 32 averages, 25 s/slice and 10 s/slice, respectively) as well as 64×64 ECG-triggered axial TOF-GRE images through the heart at the same location (4 averages, ∼1 min/slice). The coil was then tuned to ^19^F, after which the PCE ^19^F resonance frequency was determined with a pulse-acquire [11] sequence (1 shot). ^19^F GRE imaging at the same exact anatomical level was then repeated (64×64 resolution, TR/TE = 4.3/1.8 ms, 1024 averages, 4.7 min/slice).

The SNR and contrast-to-noise ratio (CNR) of a total of 33 vessels (i.e. 3 per animal) were determined in both the ^1^H and ^19^F images. The CNR was defined as (S_L_−S_S_)/N, where S_L_ is the intensity of an ROI in the lumen, S_S_ the intensity of an ROI immediately adjacent to the vessel and N the standard deviation in the background. The “pure” contrast, defined as S_L_/S_S_, was also determined for all vessels. The 33 vessels were then tracked with Soap-Bubble to determine vessel sharpness [12] (conspicuity) on both ^1^H and ^19^F images.

Two-sided, unpaired, non-equal-variance student's *t*-tests were used for statistical comparison of the quantitative findings between ^19^F and ^1^H MRA.

*In vivo* ^19^F images were post-processed for visualization with a 3-pixel-diameter Gaussian filter, fourfold cubic interpolation and thresholding at 20% of the maximum signal, while a maximum intensity projection (MIP) was generated from all multi-slice datasets.

## Results

**Figure 1:**
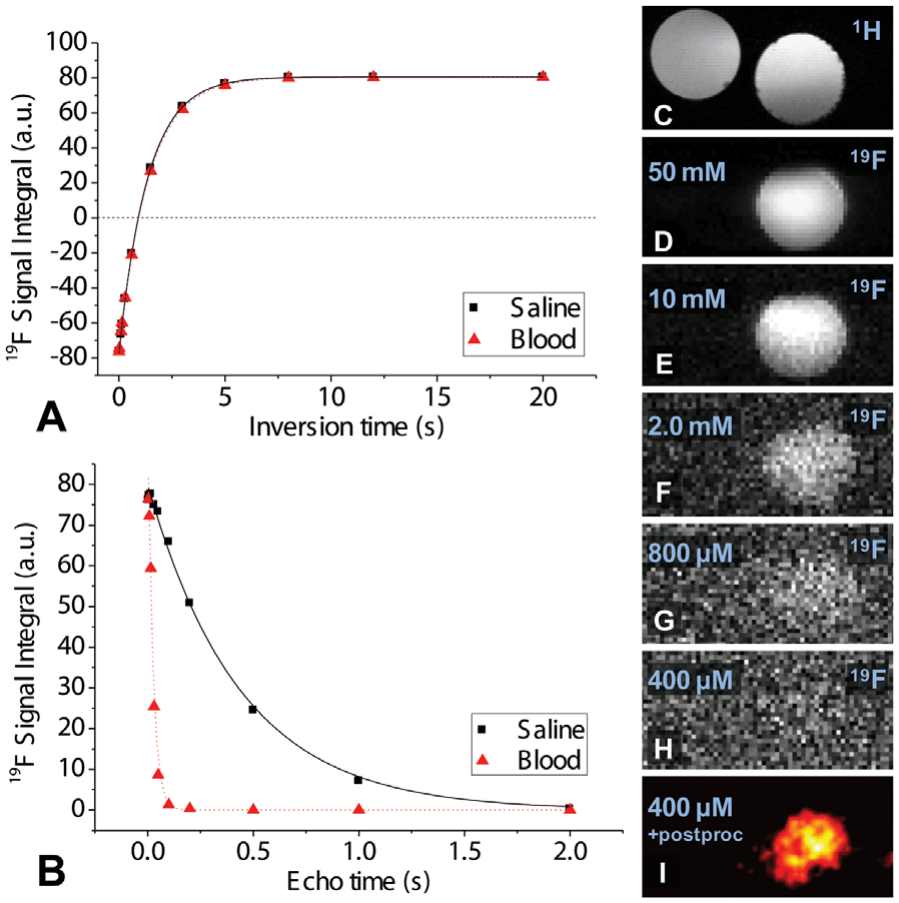
*In vitro* characterization of the PCE emulsion. **a**) ^19^F inversion recovery curve fits to determine T_1_ in saline (▪,T_1_ = 1400±30 ms) and venous blood (▴,T_1_ = 1350±40 ms). **b**) ^19^F spin-echo decay curve fits to determine T_2_ in saline and venous blood, resulting in T_2_ = 440±25 ms in saline and T_2_ = 25±2 ms in venous blood. (**c–i**) Images of dilution series in two Eppendorf tubes, one with pure saline, the other with a decreasing concentration of PCE. **c**) A ^1^H reference image, **d**) Unprocessed ^19^F MR image with signal only from the tube with 50 mM PCE; the other tube contains no ^19^F and therefore generates no signal. **e–h**) ^19^F images of different dilutions of the PCE. **i**) Postprocessed ^19^F image at the detection limit of 400 µM.

The *in vitro* ^19^F PCE T_1_ relaxation time was very similar in saline and blood (1400±30 and 1350±40 ms (Fig. 1a)), while the T_2_ was significantly reduced in venous blood from 440±25 to 25±2 ms (Fig. 1b). In the dilution series, the SNR ranged from ∼86 to ∼2 and the PCE detection limit within 10 min of scanning time was 400 µM PCE (Fig. 1c–i).

**Figure 2:**
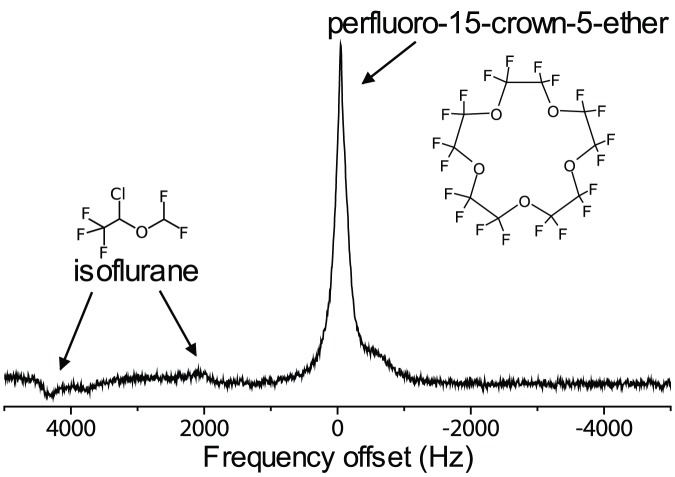
Representative unlocalized *in vivo* spectrum (single acquisition) acquired in the mouse. The crown ether singlet in the center is several times higher than and well-separated from the isoflurane resonances to the left.

In the *in vivo* spectra, the PCE's single resonance was visible with high SNR (Fig. 2). The resonances of the isoflurane anesthetic, which is known to build up in the subcutaneous fat [13], were substantially smaller.

**Figure 3:**
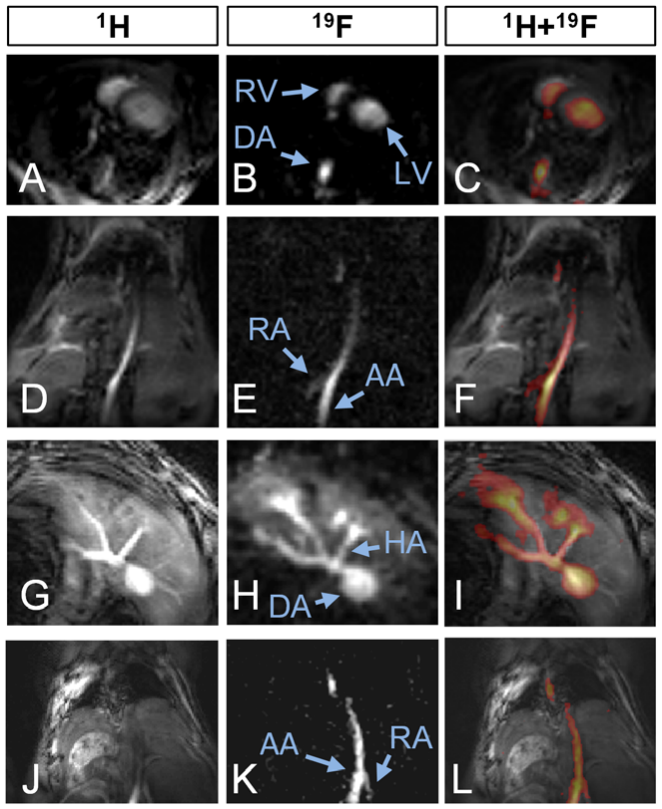
Three series of 3 *in vivo* mouse images, with ^1^H reference images in the left column, ^19^F images in the middle and a color image fusion of the two in the right column. **a–c**) axial slice through the heart, with **a** ECG-triggered to counter motion blurring, which is not needed in **b** because of the averaging (RV = right ventricle, LV = left ventricle, AA = abdominal aorta). **d–f**) Coronal slice through the lungs (dark at the top), DA and the kidneys (at the bottom), where the renal artery (RA) can be discerned. **g–i**) Axial slice at the level of the liver. In **h**, the hepatic artery (HA) and several others can be discerned while PCE is also temporarily stored in the surrounding liver tissue, thus decreasing the conspicuity of the vessels. **j–l**) Coronal slice through the abdominal aorta. Varying vessel contrast can be observed in **j**, which is not present in **k**.

The *in vivo* ^19^F MRA protocol was successfully completed in all 11 animals and exclusively visualized the blood-pool and liver at different anatomical levels (Fig. 3, middle column) while consistently coregistering with the corresponding anatomy on the ^1^H TOF images (Fig. 3, left column). The PCE appeared to be well-distributed throughout the vasculature immediately after injection and ^19^F imaging was successful until 5 hours after injection. In the axial ^19^F images at the level of the heart, both ventricles were visible and coregistered with their ECG-triggered anatomical ^1^H counterpart (Fig. 3a–c). Similar results were found on coronal slices where the iliac and renal arteries were visible within the sensitive volume of the coil (Fig. 3d–f). Slices at the level of the liver showed the celiac trunk with its three branches, while a signal increase in the liver was observed over time (Fig. 3h).

**Figure 4:**
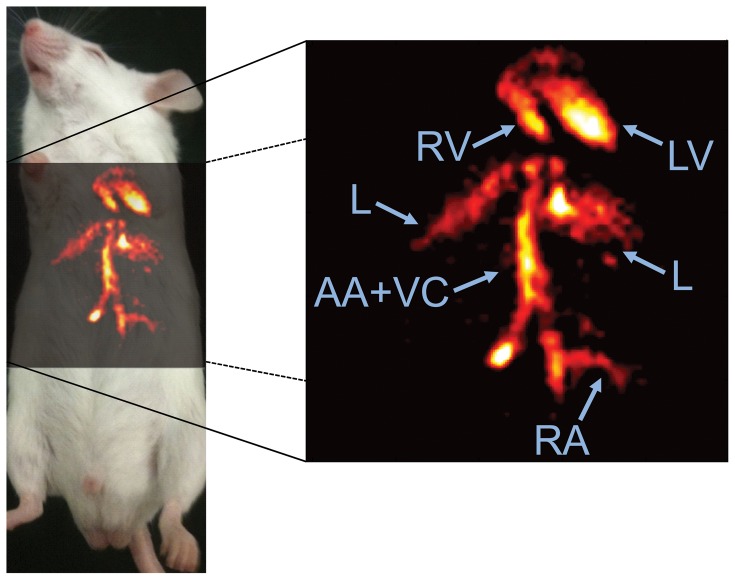
Distribution of crown ether in the vasculature of the thorax in a mouse *in vivo*, 3h after injection. Shown here is a ^19^F MRA coronal maximum intensity projection created from 6 adjacent slices with a thickness of 2mm each. The image shows the lumen of several cardiovascular structures including the right ventricle (RV), left ventricle (LV), abdominal aorta and vena cava (AA+VC), and a renal artery (RA), as well as the liver (L).

In the MIPs, long contiguous segments of thoracic and abdominal vasculature could be discerned with minimal background contamination (Fig. 4). Consistent with the spectra, no signal from isoflurane was observed in regions of subcutaneous fat.

The average SNR of the vessels was 17±8 and 83±49 (p<0.001) for the ^19^F and ^1^H images respectively, while the CNR was 15±8 and 52±35 (p<0.001). The “pure” contrast was 17±8 and 3±1 for ^19^F and ^1^H, respectively (p<0.001). With tracking, an average vessel sharpness of 66±11 and 56±12(p = 0.002) was measured in the ^19^F and ^1^H images.

## Discussion

A novel ^19^F MRA protocol for the detection of well-circulated intravascular PCE without the use of gadolinium was successfully designed, implemented and tested *in vivo*, while a quantitative analysis including a comparison with ^1^H images is provided. The GRE protocol had a similar *in vitro* detection limit as previously and *theoretically* predicted for a TSE sequence [9]. If an average human body weight of 70 kg and blood volume of 5 L are assumed, the detection limit of 400 µM used in this study is well below the dose of (1.35 g/kg)/(499 g/mol)*70 kg/5 L = 38 mM that has already been administered in humans [14], although such ^19^F MRA in humans would of course need a separate study on dose dependence. The main factors that allowed the GRE protocol to perform with similar sensitivity as TSE are most likely related to a higher number of signal averages, the use of a sensitive quadrature surface coil and the fact that TSE sequences with increased echo train length may suffer from a penalty in SNR. The measured *in vitro* ^19^F relaxation times were in agreement with the literature [15], [16] and are expected to be constant over the concentration range of PCE that can be found *in vivo*. The short T_2_ in venous blood may be explained by the presence of deoxyhemoglobin.

*In vivo*, the PCE resonance peak was more than one order of magnitude higher than those from isoflurane resonances in the spectra, indicating that the concentration of isoflurane was low when compared to that of PCE. This is consistent with the observation that there was no visible signal from fat or lungs in the ^19^F images. However, a PCE accumulation was observed in the liver, most likely due to uptake in the Kuppfer cells for clearance from the body [17].

The scanning time of ∼5 min per slice may be acceptable for *in vivo* human scanning and is consistent with the expected ∼7 h intravascular retention time of the PCE emulsion in the blood-pool [17]. However, our results were obtained on an experimental high-field animal system. In humans, the translation of this technology to lower field strengths (3T) may be necessary, but will inevitably lead to a penalty in SNR, although our injected dosage of 12 µl/g×1.78 g/ml = 0.021 g PFC/kg bodyweight is considerably lower than the 1.35 g PFC/kg bodyweight that has already been used in clinical trials [14]. In the current study we were unfortunately not able to use such doses both due to technical limitations in the production of stable emulsions with high PCE content and due to animal ethics committee limitations on administering higher volumes. It should also be noted that such large volume injections are contraindicated in patients who have coronary artery, renal, or pulmonary disease and in those who have severe hepatic disease [18]. However, with advanced and commercially available hardware (32-channel coil tunable to both ^1^H and ^19^F frequencies, parallel imaging, partial k-space sampling) on human high-field systems, imaging of one slice in ∼10 s may be feasible using contrast agent concentrations similar to our study. Furthermore, direct 3D GRE imaging will result in a similar SNR as 2D GRE imaging albeit with an increased volumetric coverage. Using a volume rather than a surface coil, the technique presented here may be extended for direct perfusion imaging as was recently partially demonstrated for the kidneys [19], since the signal can be quantified and the exact concentration determined with the addition of an external reference phantom.

The SNR of the ^19^F images was considerably lower than that of the ^1^H images (17 vs. 83), as expected and mainly caused by the lower concentration of PCE relative to water in the lumen blood-pool. The image quality is still limited, but longer scanning times, higher concentrations of contrast media or advances in hardware and software are likely to contribute to further improvement, although it has to be considered that prolonged scanning times may adversely affect image quality due to motion artifacts. The large difference in SNR between the ^1^H and ^19^F images is also the reason why the CNR is higher in the ^1^H images despite their lower “pure” contrast: the noise term appears to be more dominant than the difference in signal. However, the vessel tracking in the ^19^F and ^1^H images resulted in significantly higher vessel sharpness in the ^19^F images, most likely since the “pure” contrast between the vessel and the adjacent tissue is the dominant factor.

In conclusion, ^19^F magnetic resonance angiography of an intravenously administered perfluorocarbon emulsion supports an exclusive visualization of the vasculature *in vivo* due to the lack of background signal and provides a promising alternative, flow-independent angiographic MR technique. Since perfluoro-15-crown-5-ether is inert and non-toxic, a translation to the human setting seems feasible.
