# Correction: Fluorine-19 Magnetic Resonance Angiography of the Mouse

**DOI:** 10.1371/annotation/533f969f-0337-4145-906a-565cb11bfa8a

**Published:** 2012-08-08

**Authors:** Ruud B. van Heeswijk, Yves Pilloud, Ulrich Flögel, Jürg Schwitter, Matthias Stuber

The following information was missing from the Funding section: This work was supported by the Centre d’Imagerie BioMédicale (CIBM) of the UNIL, UNIGE, HUG, CHUV, EPFL and the Leenaards and Jeantet Foundations. 

